# Round the Hospitals

**Published:** 1920-07-03

**Authors:** 


### ROUND THE HOSPITALS.

The King held on Investiture on June 25 in the Quadrangle of Buckingham Palace and conferred decorations on the following members of the nursing services:—*The Most Excellent Order of the British Empire*: Commander, Military Division, Miss Mary Stewart, Q.A.I.M.N.S.; Member Civil Division, Miss Julia Farrer, also to receive the *Royal Red Cross, 2nd Class*. *Royal Red Cross, Bar*: Miss Elizabeth Wilson, T.F.N.S.; *First Class*: Miss Amy Fielding, Miss Alice Gilmore, Miss Katharine Lowe, Q.A.I.M.N.S.; Miss Elizabeth Downie, Miss Mabel Hobhouse, Miss Janet Livingstone,

**ROUND THE HOSPITALS**—*(continued)*.

Q.A.I.M.N.S. (R.) *Second Class*: Misses Jean Crawford, Rose Davy, Annie Falconer, Louisa Fox, Louisa Hanson, Ellen Harris, Mary Linton, Millicent Terry, Mrs. Georgina White, Q.A.I.M.N.S. (R.); Mrs. Gwendoline Arnold, Misses Julia Comyns-Berkeley, Emilie Cottle, Esther Farmer, T.F.N.S.; Misses Aimée Densham and Jane Hammick, C.N.S.; Misses Helen Anderson and Marjorie Hamilton-Dalrymple, Mrs. Gertrude Clenshaw, and Mrs. Ethel Darley, B.R.C.S.; Miss Ada Rushforth, C.H.R.; Misses Ellen Clarke, Ethel Forster, Elizabeth Gordon, Civil and War Hospitals; Misses Grace Currie, Katharine Evans, Dorothy Field, Catharine Forestal, Gwendolen Glossop, Silvia Glossop, Margaret Greatorex, Mary Hudson, Olga Nethersole, Kathleen Roberts, V.A.D. *Military Medal*: Miss Edith Hounslow, V.A.D. Queen Alexandra received all the above ladies, together with Miss A. B. Smith, R.R.C., at Marlborough House after the Investiture.

---

Queen Alexandra's Rose Day was celebrated on June 23 with an enthusiasm which was a spontaneous demonstration of popular sympathy for the Queen in her recent illness and joy in her recovery. The appeal which Her Majesty made to supporters of the movement, not in London only, but in every part of the Empire, was never more urgent, and we await with confident anticipation the news that the pressing necessities of the London hospitals have been substantially relieved by the day's festive celebrations. That the generosity of the public in the cause of the sick is by no means exhausted we have daily evidence to show. It is the sudden increase in prices that has momentarily led to a dislocation. The methods of appeal which formerly sufficed no longer keep donations up to the level of expenditure. They must be revised and a more intelligent and whole-hearted endeavour made to reach the sympathy and conscience of the people. The enthusiasm visible on Alexandra Day is an object-lesson in the power of good organisation directed to a good aim inspired by one very dear to the nation.

---

One of the happiest incidents in Alexandra Day was the appearance of Princess Arthur of Connaught at the Samaritan Free Hospital, where she visited every bed and sold roses to the much gratified patients, seventy in number, as well as to the nurses and other officials. The Princess is greatly interested in nursing and hospital work, and herself aided in the wards during the war at St. Mary's Hospital, Paddington. This is a circumstance which will not be forgotten in South Africa, whither she will shortly accompany Prince Arthur of Connaught, whose appointment by the King as Governor-General and High Commissioner of the Union of South Africa has lately been announced. Nurses in South Africa, at present a little discouraged by the tendency of the Government to legislate for them without consulting their wishes, will hail with satisfaction the advent of a Princess deeply imbued with the best traditions of the nursing services.

At the annual meeting of the Royal National Pension Fund for Nurses, the report of whose proceedings we publish on another page, the withdrawal of £86,000 owing to nurses surrendering their policies was commented on with regret. But it has been a marrying year, and the necessity for buying the house as well as the furniture which faces the newly married at present makes exceptional demands on savings. Sir Everard Hambro referred to the great services rendered to nurses by the late Sir Henry Burdett, without whom the Fund could never have been started. There was a strong desire on the part of many to commemorate his memory, and he felt that one form of commemoration might be the building up by the nurses of the Royal National Pension Fund, which Sir Henry had established and to whose work and prosperity he had continuously devoted himself throughout his life.

---

The appointment of Dame Maud McCarthy, R.R.C., to be matron-in-chief of the Territorial Force Nursing Service had been generally anticipated. Her remarkable success in the difficult office of Matron-in-Chief in France of Queen Alexandra's Imperial Military Nursing Service was due to her untiring vigilance, the confidence and loyalty she displayed towards her well-chosen group of assistants and her practical sympathy with all the nursing sisters in their onerous duties. She gained the respect of the thousands who experienced the benefits of her fine organising powers, and will find ample scope for the same powers in developing the now celebrated service of which she has become chief.

---

The Countess of Dudley, whose tragic death by drowning took place while she was bathing at Rosmuck, Connemara, on June 26, was one of the pioneers in bringing the comfort of trained nurses to the neglected homes in the west of Ireland. It was owing to her active interest in the peasantry that the Dudley nursing service was set on foot and nurses stationed in many poor centres. Lady Dudley often visited the nurses as well as the patients, and will be greatly missed by many to whom her sincerity and practical sympathy with every form of suffering had endeared her.

---

Sir Arthur Stanley, speaking at the annual meeting of the British Hospitals Association, of which he has been elected president, sounded a hopeful note in regard to the general financial condition of voluntary hospitals. He said, however, that the Forty-eight Hours Bill as it affected the working hours of nurses was a matter of concern to the Council, and forecasted a deputation from the Association to discuss the matter. The root problem is not so much finance, at least the financial difficulties can and must be met and overcome, but the scarcity of women willing to take service in the hospitals. This blocks the way to every kind of reform. To say that nurses must be relieved of the housemaid's work, for instance, is merely bringing into notice the acute shortage in th

**ROUND THE HOSPITALS**—(*continued*).

domestic staff in institutions as elsewhere. A legal forty-eight hours week imposed for nurses would make an instant enlargement of the staff necessary in every hospital and Poor-law infirmary in the kingdom, save the small minority where it is already in force. Where are the new probationers to be found?

WE offer sincere congratulations to the Incorporated Society of Trained Masseuses, which, after concentration on this aim, has at length secured its Royal Charter and must now be known under the name of the Chartered Society of Massage and Medical Gymnastics. An agreement has been entered into with the Institute of Massage and Remedial Gymnastics whereby the two societies are amalgamated under the Royal Charter and the new name. Seldom has it been granted to any society within so short a space of time so completely to alter the educational standard and professional status of its members. Sir Cooper Perry presided at the meetings held on June 19 "to celebrate the obsequies of the Society and its joyful resurrection," and the chairman of the Council, Miss Bliss, was able to announce a membership of 7,000 strong and a total of 1,000 new candidates presenting themselves for examination. The members' conference, which took place on June 24 to 26, gave evidence of the vigour and vitality which characterise this Society. The lectures and demonstrations were well attended by an enthusiastic company, the subjects were full of interest, and each was handled by an authority on the particular matter treated. We hope to deal with some of the papers in a subsequent issue.

NEVER since its foundation has the Overseas Nursing Association met under such prosperous circumstances as on June 24, when the annual meeting took place at Norfolk House. Princess Beatrice was present and Viscount Gladstone in the Chair. The story of the year's work shows a phenomenal increase in every direction. Since the first six nurses started work in 1897 their numbers have swelled to 144, nearly fifty more than in 1914 and nearly double the number at work in 1919. The attractions of the work are forcibly demonstrated by the fact that seventeen silver bars and ribands were presented during the year to matrons and nursing sisters on completing ten years and upwards good service, while twenty-two nursing sisters received silver badges on completing five years' service. The expansion of the Association continues unchecked, and calls for nursing sisters continue to come in from various parts of the world. The financial report is satisfactory, and the work is carried on at an astonishingly low cost.

WE are asked to inform all Marylebone-Infirmary-trained nurses that the Matron of St. Marylebone Infirmary invites them to a garden-party and first annual presentation of prizes on Wednesday, July 14, 1920, at 3 o'clock. Any nurses living at a distance who would like to put up for the night, and those who have not yet received an invitation-card, are asked to write to the Matron as soon as possible.

THE projected new club for nursing sisters of the Services is rapidly materialising. Temporary offices have been acquired at 35 Mecklenburgh Square, and influential support has been enlisted. The Dowager Countess of Airlie, Lady Minto, Lady Roberts, Lady Beatty, Lady Allenby, Lady Maude, and others, all officially and unofficially concerned with some branch of the nursing services, are keenly interested in the undertaking, and the professional support is unanimous and enthusiastic. Dame Ethel Becher, Miss Beadsmore Smith, Dame Sidney Browne, Dame Maud McCarthy, Miss Mickley, and many influential civilian matrons are working to consolidate the scheme, which will embrace members of Q.A.I.M.N.S., Q.A.R.N.N.S., the Reserves, T.F.N.S., the Indian Nursing Service, and possibly nursing sisters of the Ministry of Pensions' Service, and the B.R.C.S. The conditions of membership and other details have not, however, yet been decided on. If founded, as the choice of offices seems to suggest, on the other side of London from the Imperial Nurses' Club, it will assuredly not injure this admirably-conducted establishment, and will meet the needs of a very large body of nursing sisters.

PRINCESS HELENA VICTORIA, President of the Berkshire League of Mercy, was present on the 21st ult. at the opening of the famous Reading Pageant in aid of the hospitals in Berkshire and four adjoining counties. The weather was beyond reproach and some wonderful spectacular effects were obtained by the 800 performers. The Mayor, Dr. Abram, originated the idea and himself took part in the pageant as Thomas of Reading, a legendary cloth merchant. Reading (Pennsylvania) was personated by one of the Mayor's daughters, and is taking a keen interest in the doings of its mother city, having sent over a special flag for the occasion.

FEW people realise the extent of the work done under the Ministry of Pensions or the responsibilities of the nursing sisters employed in this Department under the matronship-in-chief of Miss Mary Davies. The amount of money disbursed in pensions totals £125,000,000 a year. There are 3,500,000 beneficiaries and 440 medical boards which occupy nearly 3,000 medical men in part-time service. It is estimated that one-fifth of the medical profession is engaged upon work for the Ministry. The nursing part of this great organisation is proportionately large. It has been materially strengthened by the new rate of salaries under which matrons receive £115 to £118; charge sisters, £75 to £85; nursing sisters, £60 to £65; and assistant nurses £25 to £40. War service counts towards increment in all ranks. There are vacancies in the junior ranks for which application forms can be obtained at 14 Great Smith Street, Westminster.

**ROUND THE HOSPITALS**—(*continued*).

At the nursing conference on the difficulty of staffing small hospitals, held at the Royal Horticultural Hall on June 23, Miss Ind, matron of the Stratford-on-Avon Hospital, stated that the small hospitals were handicapped because they had only low wages, long hours and lack of society to offer to their probationers. The training given counted for nothing when probationers went on to larger hospitals, and they were compelled to start afresh from the beginning. A resolution was passed calling the attention of the General Nursing Council to the present unsatisfactory position of small and special hospitals and urging the Council to consider the need for affiliation between such hospitals and the larger training-schools.

Our readers will remember that a strong protest appeared in our columns on May 1 against the action of the Borough of Bermondsey, which in advertising for a trained nurse as Health Visitor added a rider to the effect that she "will be required to belong to a trade union." We learn from the College of Nursing that Miss Rundle, at the request of the Council, brought this objectionable advertisement to the notice of the Minister of Health in the hope that he would "be able to ensure the appointment in question being thrown open to all nurses who are qualified for the post." The matter was promptly taken up by the Ministry, and a letter of remonstrance was despatched by the Ministry of Health to the Town Clerk of Bermondsey stating that "the Ministry are of opinion that a local authority should not make the terms of service of members of their staff depend upon such a condition, and they trust the Borough Council will not insist upon it." A copy of this letter was in due course forwarded to the College of Nursing, to whom the nursing world is greatly indebted for its public-spirited protest. We learn that the present members of the Bermondsey official staff who do not claim a distinct trade union belong either to the National Amalgamated Society of Co-operative Workers or to the National Association of Government Officers, and that the chief members of the staff declare themselves determined that the Council's rule shall be adhered to by all who may in future be appointed to the staff.

The presence of a member of the Board of Management in a ward in his own hospital as a paying patient is a singular test of the management. The Newark Hospital managers have recently opened an enquiry into allegations made by Mr. Lewis Ransome who was admitted as a patient at £3 3s. a week, in consequence of a serious bicycle accident and found matters very different from what he expected. His complaints were mainly of inattention and lack of skill on the part of the nurses. Mrs. Ransome, who frequently visited him, gave evidence to the same effect, and alleged irregularity and want of discipline in various directions in the hospital. Other former patients gave evidence of having suffered under bad nursing and two in particular stated they had been blistered by hot-water bottles. For the defence it was shown that Mrs. Ransome had openly talked in an injudicious manner of the matron, and many of the allegations were denied. The enquiry was adjourned. The hospital contains thirty-five beds and is staffed normally by the matron, three sisters, two staff-nurses, and seven probationers.

*The Times* heralds its new *Woman's Supplement* prettily enough on its page cover with the coloured presentment of a Titian-haired lady in early Victorian costume, symbolical of "Late June." The contents are in keeping with this fragrantly feminine creature, who ushers the reader into the details of a handsomely appointed, well-illustrated publication. The aim is rather towards gratification of the eye than mental stimulus, for the abundance and variety of pictures outweigh the letterpress. Whether in photography, line and wash sketches or colour print this issue is rich in visions of which the greater portion are applied to fashion and the art of the toilette. The scope of this fortnightly magazine, however, promises to be wide, covering travel, art in its many forms, politics, sport, education, in addition to the essentially feminine themes of dress, cuisine and health.

Appropriately to "Rose Day" (its birthday) *The Times Woman's Supplement* contains a leading article on Queen Alexandra by Mrs. Stuart Menzies, written not so much from the public side as that of the personality of the Queen in her private life at Sandringham. In "A Quiet Hour" Mr. Polygon Amor reviews breezily the educational system of William Platt apropos of the work by that very original expert on child-training, "The Joy of Education." George Duncan brings his valuable experience to bear on "The Lady Golfer." Hints on the training of women as public speakers may be culled from an article in which Mme. d'Esterre and Miss Macarthy give their views, which are accentuated by a companion article, "Six months in Parliament," by Lady Astor. Lady Astor emphasises both the anxiety of man members to ascertain the woman's viewpoint, and the need for women members. Women, she considers, have absorbed the new post-war spirit—the sense of sacrifice and brotherhood—more completely than men, and are therefore urgently needed in public life to accelerate the work of reconstruction.

Lambeth Council Health Committee proposes equality of men and women sanitary inspectors. This is the result of a communication from the Women Sanitary Inspectors and Health Visitors Association which urged that there should be equal pay for men and women in proper health departments, working side by side. The report expresses agreement with the claim made by the Association, stating that in view of the fact that women inspectors have to undergo the same professional training as men, and to pass the same qualifying examination, they, and also the health visitors—who also have to be certified—should be paid the same salary, namely, £350 per annum, rising by six annual increments of £15 and one increment of £10 to a maximum of £450 per annum.

